# Abortive and Prophylactic Therapies to Treat Migraine in Pregnancy: A Review

**DOI:** 10.7759/cureus.70807

**Published:** 2024-10-04

**Authors:** Mohammed O Ibrahim, Dana Sarmini

**Affiliations:** 1 General Practice, College of Medicine, Mohammed Bin Rashid University of Medicine and Health Sciences, Dubai, ARE; 2 Pediatrics, Al Jalila Children's Speciality Hospital, Dubai, ARE

**Keywords:** abortive and prophylactic therapies, headache, migraine, migraine in pregnancy, migraine treatment in pregnancy

## Abstract

Migraine is a common issue during pregnancy, often affected by hormonal changes. More than half of the women affected by migraine experience improvement in or remission of migraine symptoms, particularly during the second and third trimesters, with those having menstrual migraines or migraines without aura benefiting the most. However, a small percentage of women may see a worsening of their migraines, especially those with migraine with aura, and some may even develop migraines for the first time during pregnancy, often in the first trimester. Postpartum, many women experience a recurrence of migraines, likely due to the drop in estradiol and endorphin levels. A literature search was performed in PubMed for articles published from 2013 through 2023, and 80 out of 362 publications were included. When it comes to managing pregnant women with migraine, non-pharmacological treatments are preferred, including lifestyle modifications and avoiding known triggers. When medication is necessary, acetaminophen is the first-line treatment, with nonsteroidal anti-inflammatory drugs and triptans regarded as secondary options, though trimester-specific risks limit their use. Preventive treatments, if required, may include low doses of β-blockers or amitriptyline but should be used cautiously. This article aims to provide a concise overview of the existing research on the acute and prophylactic use of medications to treat migraines in pregnant and lactating women. Furthermore, it presents recommendations for healthcare professionals managing pregnant females presenting with migraine in clinical settings.

## Introduction and background

Migraine is a prevalent neurological disorder among women of reproductive age, with a higher incidence in the fourth decade of life and a one-year prevalence of approximately 25% [1]. Throughout various stages of life, including menarche, pregnancy, menstrual cycles, use of hormonal therapies, and menopause, hormonal fluctuations can significantly impact the frequency and severity of migraine attacks [2]. For instance, during pregnancy, migraine attacks may worsen in the first trimester due to hyperemesis gravidarum symptoms but often improve in the second and third trimesters as estrogen levels rise [3]. During pregnancy, a significant percentage of women with migraine experience an improvement in symptoms, with up to 80%-90% showing progressive relief and some even achieving complete remission [4]. Despite the improvement during pregnancy, more than half of these women may see a return to pre-pregnancy migraine levels within a month after delivery, often exacerbated by factors like sleep deprivation and stress [5]. Many women are concerned about how migraine might affect their pregnancy, with 20% of them avoiding pregnancy due to fears that their migraine condition could worsen, complicate the pregnancy, or negatively impact their child [5]. The impact of breastfeeding on preventing migraine recurrence post-delivery is still debated, with conflicting evidence on its protective effects [6].

Non-pharmacological approaches are essential for managing various conditions during pregnancy. These approaches, such as valsalva maneuvers, progressive muscle relaxation, music therapy, aerobic exercise, massage, and maternity support belts, are safe and potentially effective strategies that should be considered first-line treatments and prioritized as first-line treatments [7,8]. Additionally, ensuring adequate nutrition, sleep, exercise, and utilizing biofeedback and relaxation methods are recommended; focusing on enhancing overall well-being may lead to a potential reduction in the necessity for medication [8]. In the context of a cohort study involving a group of 3,480 female individuals who experienced migraine episodes while pregnant, it was observed that a substantial majority, exceeding 70%, acknowledged the use of pharmacological interventions specifically targeting migraines, among which non-opioid analgesics accounted for 54% of the cases. At the same time, triptans were administered in 25% of the instances as the most commonly employed therapeutic agents [9]. Medication use is even more prevalent among breastfeeding women with migraine, highlighting the challenge of managing severe and debilitating migraine attacks during this period [9].

The safety of drug therapy for migraine during pregnancy is a complex issue, as obtaining high-quality safety data is challenging due to ethical considerations and the reliance on observational studies that often lack specificity [10]. Treatment of migraine during pregnancy requires consideration of the safety profile of medications, with some medications like nonsteroidal anti-inflammatory drugs (NSAIDs) considered relatively safe during the second trimester but used with caution, while others like ergot derivatives and calcitonin gene-related peptide (CGRP) antagonists avoided [11]. Non-pharmacological techniques are recommended as the first line of treatment, with a medication like acetaminophen considered an effective option for transient migraine attacks during pregnancy or breastfeeding [11]. Suboptimal management of migraine occurring in pregnant women can undeniably result in adverse outcomes for not only the expecting mother but also the developing fetus, including compromised nutritional intake, inadequate fluid balance leading to dehydration, chronic lack of restful sleep contributing to sleep deprivation, elevated levels of psychological stress, and heightened susceptibility to experiencing symptoms of clinical depression [12].

Several studies have highlighted the relationship between migraine during pregnancy and an increased risk of vascular complications such as stroke, preeclampsia, and gestational hypertension [12,13]. For instance, a history of migraine before pregnancy has been associated with a higher risk of hypertensive disorders of pregnancy, with the most significant risk observed among those whose migraines persisted into the first trimester [14]. Additionally, pre-pregnancy migraine has been associated with higher risks of preterm delivery, gestational hypertension, and preeclampsia, with the risk of preeclampsia being somewhat higher among those with migraine with aura in contrast to those without aura [15]. These complications have the potential to result in severe consequences, including premature birth, infants born smaller than expected for their gestational age, and separation of the placental abruption [16]. Managing migraine with medications while pregnant and during the postpartum period can be difficult because of the potential risks to the fetus and newborn [17].

Many widely prescribed drugs do not have documented safety information regarding their use during pregnancy or breastfeeding [18]. Modifying established therapeutic regimens may be necessary to identify safer options [19]. Through this review article, we aim to provide a concise overview of the existing research on the acute and prophylactic use of medications to treat migraines in pregnant and lactating women. Recommendations for healthcare professionals managing pregnant females presenting with migraine in clinical settings are also presented.

## Review

Methods

A literature search was performed in PubMed for English-language articles published from 2013 through 2023 using terms and keywords such as ‘migraine’, ‘headaches’, ‘migraine in pregnancy’ and ‘migraine treatment in pregnancy’, and 362 publications were found. Out of these 362 publications, 80 were included, consisting of 30 observational studies, 21 review articles, 10 systematic reviews, 5 book chapters, 4 editorials, 3 guidelines, 3 experimental studies, 1 clinical trial, 1 population registry study, 1 invited speaker presentation, and 1 commentary. This article comprehensively reviewed the most reliable and highest quality evidence on the topic, ensuring the inclusion of references frequently mentioned and cited in guidelines and previous review articles. The remaining 282 publications were excluded as they were irrelevant to the study aim and had less study citations. This review aligned with the migraine treatment guidelines from the American Academy of Neurology and the American Headache Society.

Management of migraine during pregnancy

Management of migraine during pregnancy is done using acute and prophylactic medications. Acute or abortive therapy includes pain control and nausea/vomiting control. Pain control medications include acetaminophen, NSAIDs, triptans, opioids, and ergot derivatives. Nausea/vomiting control medications include antiemetics, including diphenhydramine, promethazine, metoclopramide, chlorpromazine, and ondansetron.

Abortive therapies

Acetaminophen

Acetaminophen is widely regarded as a safe option for managing migraines during pregnancy. It is typically advised as the first-line treatment due to its minimal teratogenic risk compared to other medications like NSAIDs and opioids, which are generally avoided during pregnancy [20-23]. Acetaminophen has been widely used during pregnancy for its analgesic and antipyretic properties. Dumitru et al. showed that acetaminophen is effective in alleviating mild to moderate migraine symptoms when used alone or in combination with antiemetics [24]. While some studies have indicated a possible association between long-term prenatal acetaminophen exposure and adverse neurodevelopmental outcomes in children, including an increased risk of conditions like attention-deficit/hyperactivity disorder (ADHD), other studies have not definitively established this association [25]. The European Medicines Agency (EMA) has reviewed these results and considers the proof insufficient to validate a direct connection between exposure to acetaminophen during pregnancy and its impact on neurodevelopment [26]. A high cumulative use of acetaminophen during pregnancy, especially in the highest quintile of exposure, has been associated with a modestly increased risk of preeclampsia. This emphasizes the significance of monitoring dosage and duration [27]. While acetaminophen remains a cornerstone in the treatment of migraines during pregnancy, its usage should be carefully evaluated, balancing efficacy with potential risks. Therefore, acetaminophen remains a commonly recommended and first-line treatment option for pain relief during pregnancy, with its use considered safe for mild to moderate migraine [28].

NSAIDs

Nonsteroidal anti-inflammatory drugs are commonly used for analgesia, but their use during pregnancy is controversial due to potential risks to both the fetus and the mother. Choi et al. showed that NSAID use in early pregnancy is associated with slightly increased risks of major congenital malformations (MCMs), low birth weight, and oligohydramnios, though not antepartum hemorrhage [29]. The cyclooxygenase (COX) pathway, which NSAIDs inhibit, plays a crucial role in tissue inflammation and developmental processes, and its perturbation can lead to developmental defects [30]. NSAID use in the third trimester is generally advised against due to risks like ductus arteriosus constriction and oligohydramnios, especially with prolonged use [31]. Despite these risks, NSAIDs are still prescribed during pregnancy, with ibuprofen being the most common, followed by diclofenac and naproxen [32]. There is also evidence suggesting an increased risk of miscarriage with NSAID use around the time of conception, though not necessarily during the first trimester [33]. Additionally, periconceptional NSAID use may increase the risk of neural tube defects like spina bifida, particularly in women with inadequate folic acid intake [34]. NSAIDs, including indomethacin, can impact pregnancy by delaying delivery and influencing fetal outcomes depending on the timing and duration of exposure [35]. In neonates, NSAIDs have been considered for analgesia and managing conditions like patent ductus arteriosus, but concerns exist regarding their effects on prostaglandin levels and organ development, particularly, the kidneys [36]. Overall, while NSAIDs can be effective for pain management, their use during pregnancy should be carefully considered, weighing the benefits against potential risks, and alternative treatments should be explored where possible.

Triptans

Triptans, a class of tryptamine-based drugs used to treat migraines, have been studied for their safety during pregnancy with mixed results. The use of triptans for migraine treatment in pregnancy is a complex topic due to the potential risks and benefits involved. While some data suggest that certain triptans, particularly sumatriptan, may be safe, the consensus is that they should be used cautiously and only when the benefits outweigh the risks [37]. While no significant increase in MCMs has been found in most studies, there are concerns about other adverse outcomes [38]. For instance, a cohort study found no significant increase in MCMs or prematurity but did report a higher rate of spontaneous abortions in triptan-exposed pregnancies [39]. Similarly, a study using the Norwegian prescription database found no association between triptan use and congenital malformations but did note an increased risk of postpartum hemorrhage [40]. Also, a meta-analysis reported cases of fetal death and intrauterine growth retardation, suggesting that vasoconstrictive properties of triptans might be responsible for these outcomes [41]. A study by Yusuf et al. also indicated no increased risk of major congenital disabilities or spontaneous abortions compared to non-migraine cohorts [42]. In addition, a study found evidence of a slight increase in the risk of uterine atony and hemorrhage during the second and third trimesters [43]. Despite these findings, the use of triptans during pregnancy should be carefully considered, especially given the potential for increased rates of spontaneous abortions and other complications [44]. Overall, while triptans do not appear to be a major teratogen, their use during pregnancy should be weighed against potential risks, and further research is warranted to confirm these findings [45].

Opioids

Opioid use during pregnancy is a notable public health concern associated with numerous adverse maternal and neonatal outcomes. Studies have shown that opioid use in pregnancy can lead to increased risks of cardiac arrest, placental insufficiency, neonatal intensive care unit admission, preterm birth, and small for gestational age infants [46]. The prevalence of opioid use among pregnant women has been increasing, with many women using prescription opioids for pain management or opioid dependency [47]. Despite the risks, opioid maintenance therapies like buprenorphine and methadone are recommended for pregnant women with opioid use disorder (OUD) due to their effectiveness in reducing illicit drug use and improving pregnancy outcomes, although they are associated with neonatal abstinence syndrome (NAS) [48]. In addition, the use of opioids is also associated with higher rates of neonatal respiratory depression and cesarean sections [49]. Extended use should be explicitly discouraged while pregnant; however, in cases of brief and intermittent use, weak opioids like codeine could serve as a viable alternative in situations where other medications have proven ineffective. The need for standardized guidelines to manage migraines in pregnancy is evident, as current practices vary widely and may not always prioritize fetal safety [50]. Therefore, given the complex interplay of risks and the need for effective management, it is essential to continue research and develop targeted interventions to mitigate the side effects of opioid use during pregnancy and enhance maternal and fetal health outcomes.

Ergot Derivatives

Ergot alkaloids, derived from the fungus *Claviceps purpurea*, have historically been used in obstetrics to control excessive uterine bleeding and other gynecological conditions, but their use in pregnancy is fraught with significant risks [51]. Ergot derivatives, such as ergotamine, are known to induce severe vasoconstriction, which can lead to acute myocardial infarction and other cardiovascular complications, even in healthy individuals, and have been implicated in fatal postpartum cases [51]. Clinical reports have also documented severe outcomes such as gangrene of the bowel in pregnant women, suggesting a vasospastic mechanism [52]. While some studies have highlighted the teratogenic risks associated with various drugs, a prospective study showed that there was no significant increase observed in major malformations with ergot usage. However, there was an increased risk of preeclampsia and preterm birth when these drugs were used later in pregnancy [53]. While initial concerns suggested that ergots might cause congenital malformations due to vascular disruption, cohort studies and data from Swedish health registers have not confirmed these teratogenic effects [54]. However, a study on cabergoline, another ergot derivative, showed no increase in miscarriage rates or congenital malformations, suggesting some ergot derivatives might be safer than others [55]. Although ergot derivatives have displayed significant effectiveness in the management of migraines, their use in pregnancy is strongly not recommended due to potential risks posed to both the fetus and the mother. The absence of teratogenic effects in certain studies does not adequately mitigate the risks associated with uterine contractions and vascular complications. Overall, the evidence strongly advises against the use of ergot alkaloids during pregnancy due to their potential for severe maternal and fetal complications.

Antiemetics

Antiemetics play a vital role in managing migraines in pregnancy. During pregnancy, treatment choices are restricted and limited due to possible hazards to the fetus. The selection of antiemetics must balance efficacy in alleviating nausea and vomiting associated with migraines while assuring safety for both the mother and the developing fetus. During pregnancy, managing migraines, particularly those accompanied by nausea and vomiting, requires careful selection of antiemetics to ensure both maternal and fetal safety. Ondansetron is frequently recommended as the antiemetic of choice for pregnant women, including those experiencing migraines. It is considered effective in relieving nausea and vomiting and is regarded safe during pregnancy, although some studies suggest caution due to potential risks [56,57]. Metoclopramide, a dopamine antagonist, is effective in treating nausea associated with migraines and is often used in conjunction with oral anti-migraine agents, as it enhances the response to oral anti-migraine agents. It is typically regarded safe for use in pregnancy [58]. Another dopamine antagonist, prochlorperazine, is used to relieve nausea and other migraine symptoms. It is considered safe for use during lactation and may be used during pregnancy with caution [58]. For mild cases, acetaminophen combined with antiemetics is often the first line of treatment, while more severe cases may necessitate the use of triptans, albeit with caution due to potential risks [59]. While antiemetics like ondansetron and metoclopramide are commonly used to manage migraine-associated nausea during pregnancy, the selection of therapy should be individualized, considering the severity of symptoms and potential risks. In conclusion, the choice of antiemetics should be guided by the severity of symptoms, possible risks to the fetus, and the effectiveness of non-pharmacological interventions, ensuring a balanced approach to migraine management during pregnancy.

Prophylactic therapies

Beta-Blockers

Beta-blockers constitute a pharmacological category frequently employed for the prophylaxis of migraines, and their administration during pregnancy introduces distinct considerations. Though hormonal variations usually lessen migraine occurrence in pregnancy, certain women still experience severe migraines that could call for therapeutic measures. The safety and effectiveness of beta-blockers in the context of migraine prophylaxis among pregnant individuals are contingent upon both the prospective advantages in alleviating migraine frequency and the associated risks linked to fetal exposure to these pharmacological agents. Beta-blockers are commonly used for migraine prophylaxis and have shown efficacy in reducing the frequency, duration, and intensity of migraine attacks [60]. Propranolol, metoprolol, and atenolol are the beta-blockers effective in migraine prophylaxis [61]. Labetalol, a beta-blocker, has also been used successfully in pregnant women with preeclampsia to manage migraine without significant side effects [61]. Propranolol, in particular, has been extensively studied and proven effective in numerous controlled trials for migraine prevention [62]. Despite their benefits, there are concerns about the potential teratogenic effects of beta-blockers, although they are one of the most commonly used types of medication for managing high blood pressure during pregnancy [63]. Research has not decisively linked beta-blocker intake to congenital anomalies; nevertheless, caution is recommended, particularly in the first trimester of pregnancy [64]. Although beta-blockers show efficacy in migraine prophylaxis, their use during pregnancy merits careful evaluation. The use of non-pharmacological methods and lifestyle modifications are advocated to decrease fetal exposure to drugs. A detailed review of risks and benefits is essential for patients where medication is crucial. Similarly, migraine relief during pregnancy in many women indicates that treatment may not be necessary during that period, therefore highlighting the significance of personalized treatment regimens for each patient [65]. Overall, while beta-blockers can be a viable option for migraine prophylaxis during the second and third trimesters, their use should be carefully considered and monitored to balance efficacy and safety for both the mother and the fetus [65].

Cyproheptadine

Cyproheptadine, a first-generation H1 antihistamine, is considered a safe option for migraine management during pregnancy [66]. It has been used for decades for migraine prevention and is deemed safe for use in pregnancy, offering an inexpensive alternative for pregnant women suffering from migraines [67]. The drug is effective in reducing the frequency, duration, and intensity of migraine attacks [68]. Despite its benefits, cyproheptadine is associated with side effects such as weight gain and fatigue, which may limit its appeal to some patients [69]. However, it is crucial to note that cyproheptadine is not recommended for use during breastfeeding due to potential risks [69]. While cyproheptadine is effective, it is not the only option. Other treatments, such as lifestyle modifications and non-pharmacological interventions, should be considered first to minimize drug exposure during pregnancy [69]. In summary, cyproheptadine presents a viable option for managing migraines during pregnancy, providing a safe and cost-effective alternative for expectant mothers experiencing migraine symptoms, but caution should be exercised during breastfeeding.

Calcium Channel Blockers

Calcium channel blockers (CCBs) have shown efficacy in migraine prophylaxis, including during pregnancy. CCBs have been documented to have a prophylactic effect on migraines, reducing the frequency, severity, and duration of attacks; however, the optimum effect is often observed after more than two months of treatment [70]. By blocking calcium influx, CCBs can reduce migraine attacks' frequency, severity, and duration [70]. Nimodipine, a selective cerebral vasculature CCB, has been particularly effective in decreasing migraine frequency and duration [71]. A multicenter observational study found no significant increase in major birth defects with CCB use during the first trimester, although there was a higher incidence of preterm births and lower birth weights, likely due to underlying conditions rather than the medication itself [71]. While CCBs present potential advantages for the prophylaxis of migraines and certain pregnancy-associated conditions, their application during pregnancy necessitates a meticulous evaluation of possible risks. The absence of teratogenic effects is reassuring; however, the implications in relation to neonatal birth weight and the rates of preterm delivery warrant a prudent approach. Additional investigations are imperative to delineate their safety profile comprehensively and to refine their application in pregnant individuals experiencing migraines. In conclusion, CCBs could be a viable option for managing migraines in pregnant individuals, offering both efficacy and safety benefits.

Antidepressants

The use of antidepressants for migraine treatment in pregnant patients involves a complex problem that merits an in-depth examination of the therapeutic outcomes and the risks to fetal health. Despite the effectiveness of antidepressants, especially tricyclic antidepressants like amitriptyline, in managing migraine prevention, their administration during pregnancy needs a careful approach. Antidepressants like amitriptyline have shown efficacy in migraine prophylaxis; amitriptyline is widely recognized for its effectiveness in migraine prophylaxis. It is often recommended as a primary treatment due to its efficacy in reducing migraine frequency and severity, especially when other treatments have not been effective [29]. A meta-analysis of randomized controlled trials showed that antidepressants, particularly amitriptyline, significantly reduce migraine frequency, when compared to placebo, although they are associated with a higher incidence of adverse effects [72]. Also, studies have shown that selective serotonin reuptake inhibitors (SSRIs) like venlafaxine are not more effective than placebo or amitriptyline in reducing migraine frequency, intensity, and duration over two to three months of treatment [73-75]. Additionally, maternal SSRI use during pregnancy has been associated with higher risks of gestational hypertension and preeclampsia, with a relative risk of 4.9 for preeclampsia if SSRI treatment is continued beyond the first trimester [76]. The safety of antidepressants during pregnancy is a critical concern; while amitriptyline is considered relatively safe at lower doses, its use should be carefully monitored to minimize potential risks to the fetus [77]. In addition, the use of antidepressants during pregnancy has been associated with risks such as postnatal adaptation syndrome and persistent pulmonary hypertension in the newborn, although untreated depression also poses significant risks [78]. While SSRIs may be more tolerable than tricyclic antidepressants, their efficacy in preventing migraines during pregnancy is not supported by high-quality evidence, emphasizing the need for careful consideration of their use in pregnant individuals suffering from migraines [78].

Gabapentin

Gabapentin, a medication commonly used for epilepsy, neuropathic pain, and migraine prophylaxis, has been a subject of concern regarding its safety during pregnancy [79]. A large population-based study analyzing the US Medicaid data found no significant association between taking gabapentin in early pregnancy and major birth defects [79]. Some clues indicated a higher risk of heart anomalies associated with greater exposure levels. Studies have shown that gabapentin exposure during pregnancy may not significantly increase the risk of major malformations but could potentially lead to a higher risk of cardiac defects, preterm birth, and neonatal intensive care unit admissions [80]. Additionally, gabapentin has been flagged as potentially "developmentally toxic" with unknown risks of birth impacts. Concerns have been raised about atypical withdrawal symptoms in neonates exposed to gabapentin in utero, especially when combined with opioids [80,81]. In terms of effectiveness, clinical trials have generally not supported the use of gabapentin for migraine prophylaxis. A Cochrane review concluded that gabapentin is not effective for preventing episodic migraines in adults, with common adverse effects such as dizziness and somnolence [82]. Given these findings, caution is advised when considering gabapentin use in pregnant individuals, especially in the context of migraine treatment, as further research is required to thoroughly understand the risks and benefits of gabapentin exposure during pregnancy.

## Conclusions

Migraine treatments during pregnancy present a complex issue as a result of the limited availability of safety information and concerns regarding potential harm to the developing fetus. Although the majority of expectant mothers with migraines see improvement in their symptoms, some may encounter substantial disability, particularly in the first trimester. The utilization of migraine medications while pregnant, mainly when used daily and involving certain types such as preventive treatments or a combination of analgesia and caffeine, is linked to a higher likelihood of spontaneous miscarriage. This emphasizes the importance of understanding how these medications affect pregnancy outcomes. Therefore, nonpharmacological approaches are typically the first line of treatment for managing mild migraine and are recommended to be utilized in conjunction with pharmacological therapy when necessary. In addition, prophylactic treatment should solely be contemplated for the most extreme cases. Therefore, in abortive therapies, acetaminophen, and in preventive treatment, beta-blockers and CCBs are first-line treatments.
